# Deep Learning Models to Predict Fatal Pneumonia Using Chest X-Ray Images

**DOI:** 10.1155/2022/8026580

**Published:** 2022-11-24

**Authors:** Satoshi Anai, Junko Hisasue, Yoichi Takaki, Naohiko Hara

**Affiliations:** Division of Respiratory Medicine, Iryo Hojin Harasanshin Byoin 1-8, Taihaku-Cho, Hakata-Ku, Fukuoka 812-0033, Japan

## Abstract

**Background and Aims:**

Chest X-ray (CXR) is indispensable to the assessment of severity, diagnosis, and management of pneumonia. Deep learning is an artificial intelligence (AI) technology that has been applied to the interpretation of medical images. This study investigated the feasibility of classifying fatal pneumonia based on CXR images using deep learning models on publicly available platforms.

**Methods:**

CXR images of patients with pneumonia at diagnosis were labeled as fatal or nonfatal based on medical records. We applied CXR images from 1031 patients with nonfatal pneumonia and 243 patients with fatal pneumonia for training and self-evaluation of the deep learning models. All labeled CXR images were randomly allocated to the training, validation, and test datasets of deep learning models. Data augmentation techniques were not used in this study. We created two deep learning models using two publicly available platforms.

**Results:**

The first model showed an area under the precision-recall curve of 0.929 with a sensitivity of 50.0% and a specificity of 92.4% for classifying fatal pneumonia. We evaluated the performance of our deep learning models using sensitivity, specificity, PPV, negative predictive value (NPV), accuracy, and F1 score. Using the external validation test dataset of 100 CXR images, the sensitivity, specificity, accuracy, and F1 score were 68.0%, 86.0%, 77.0%, and 74.7%, respectively. In the original dataset, the performance of the second model showed a sensitivity, specificity, and accuracy of 39.6%, 92.8%, and 82.7%, respectively, while external validation showed values of 38.0%, 92.0%, and 65.0%, respectively. The F1 score was 52.1%. These results were comparable to those obtained by respiratory physicians and residents.

**Conclusions:**

The deep learning models yielded good accuracy in classifying fatal pneumonia. By further improving the performance, AI could assist physicians in the severity assessment of patients with pneumonia.

## 1. Introduction

Pneumonia is a leading cause of morbidity and mortality globally. In 2019, it caused 1.23 million deaths in adults older than 70 years and 2.49 million deaths in persons of all ages globally [1]. In Japan, pneumonia is classified mainly into community-acquired pneumonia (CAP), nursing and healthcare-associated pneumonia (NHCAP), and hospital-acquired pneumonia (HAP). We have previously reported the relationship between spleen volume and severity and mortality in patients with pneumococcal pneumonia [2]. Chest X-ray (CXR) is indispensable to the assessment of the severity and diagnosis of pneumonia [3]. The radiographic features of bilateral shadows, involvement of more than one lobe, bilateral pleural effusions, or the presence of a cavity predict a worse prognosis in pneumonia [4, 5]. Therefore, the diagnosis and assessment of pneumonia severity from CXR images is important, but it is not performed accurately by nonrespiratory specialist physicians [6]. Deep learning is a technique of machine learning in artificial intelligence (AI) technology, [7] using artificial neural networks as computational models to discover intricate structures and patterns in large, high-dimensional datasets [7]. ImageNet, a large dataset of more than 14 million human-annotated images, has been instrumental in the development of deep learning in image recognition. Classification errors in the annual ImageNet's large-scale visual recognition challenge have decreased more than eightfold over the past 6 years, to less than 3% in 2017, surpassing human performance [8]. Advances in deep learning and the availability of digitized healthcare data have contributed to a growing number of studies describing deep learning applications in the field of medical imaging, such as chest radiographs [9]. Specifically, deep learning algorithms can differentiate normal CXR images from those showing pneumonia and diagnose pneumonia accurately with a sensitivity of 81–100% and a specificity of 56.6–100% [10–15]. In addition, since the global pandemic of coronavirus disease 2019 (COVID-19), some deep learning models have been developed to diagnose COVID-19 pneumonia using CXR images, with a sensitivity of 71–98.8% and specificity of 90–92.9% [16–20]. Furthermore, studies of a deep learning model using CXR images to assess the prognosis and severity of COVID-19 pneumonia have been reported. Cohen et al. developed a deep learning algorithm to predict the severity of COVID-19 pneumonia using CXR images [21]. Zhu et al. developed a deep learning model to assess the severity of COVID-19 infection [22]. Recently, Li et al. have developed a deep learning Siamese network to predict the radiographic assessment of lung edema (RALE) scores used to assess the severity of acute respiratory distress syndrome in patients with COVID-19 [23]. However, to the best of our knowledge, the prognosis prediction of non-COVID-19 pneumonia by deep learning using CXR images has not been sufficiently studied. In the era of the COVID-19 pandemic, the number of deaths due to pneumonia remains high. Hence, the development of prognostic tools for pneumonia patients is vital, and computer-aided diagnosis techniques based on deep learning can be used as a supplement in the clinical decision-making process. We performed a study to establish an AI diagnostic tool for assessing the fatality of pneumonia using CXR images with deep learning models.

## 2. Methods

### 2.1. Patients and Dataset

**Figure 1:**
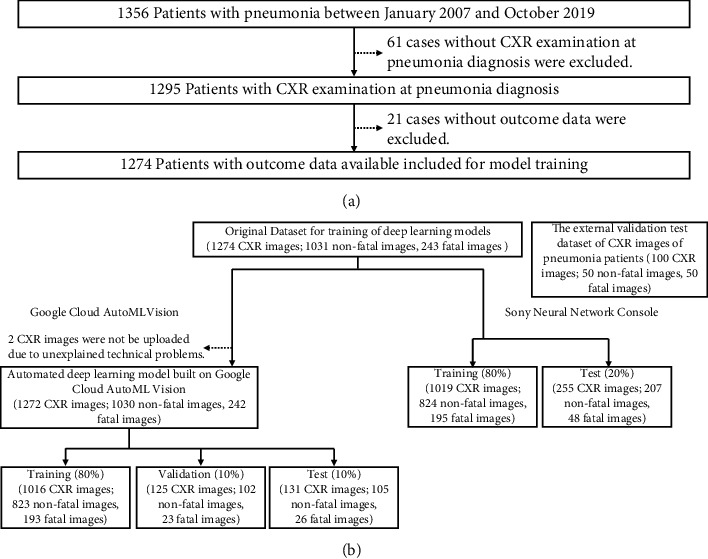
Patient selection flow. (a) Flowchart demonstrating the exclusion and inclusion of patients with pneumonia at Harasanshin Hospital. (b) Splitting of the CXR image datasets at pneumonia diagnosis for training and testing of the deep learning model using Google Cloud AutoML Vision and Sony Neural Network Console. CXR, chest X-ray.

We retrospectively investigated patients with pneumonia who underwent CXR examination at diagnosis in the Department of Respiratory Medicine at Harasanshin Hospital, between January 2007 and October 2019. We then created a CXR image original dataset of patients with pneumonia at diagnosis for deep learning modeling (Figure 1(a)). No patient with COVID-19 pneumonia was included in this cohort. The diagnostic criteria for pneumonia are listed in Table S1. Microbiological diagnosis was performed using cultures (sputum, blood, bronchial wash, and pleural effusion). Fatal cases were defined as cases of patients who died from pneumonia at Harasanshin Hospital, while nonfatal cases were defined as cases of patients who recovered from pneumonia following outpatient treatment or inpatient treatment and were discharged from Harasanshin Hospital. Complications of congestive heart failure (CHF) have been reported to affect the diagnosis and prognosis of pneumonia [24]. Therefore, we evaluated the complications associated with CHF. Patients with pneumonia and CHF complications were defined as those diagnosed with chronic CHF or new heart failure at the time of pneumonia diagnosis. The diagnostic criteria for new heart failure are listed in Table S2. Furthermore, we prepared an external validation test dataset of 100 CXR images (50 CXR images of patients with fatal pneumonia and 50 CXR images of patients with nonfatal pneumonia who were mainly treated in the Department of General Internal Medicine at Harasanshin Hospital and not used in the training of the deep learning models) (Figure 1(b) and Table S3) to externally validate the performance of deep learning models. The requirement for written informed consent was waived because of the retrospective observational approach, and the study was carried out using the opt-out method based on our hospital website. The study was performed in accordance with the Declaration of Helsinki and approved by the Institutional Review Board of Harasanshin Hospital (No. 2020-09, May 5, 2020). The datasets were not publicly available for legal and ethical reasons. We retrospectively collected the following data from the medical records of the patients: background characteristics, laboratory test findings at the onset of pneumonia, physical examination findings, CXR findings, and clinical courses.

### 2.2. Image Preparation and Model Training

CXR images of pneumonia patients at diagnosis were evaluated for the cardiothoracic ratio (CTR), [25] the number of lobes involved with infiltrate (1 or ≥2), the location of infiltrate (unilateral or bilateral), the location of pleural effusions (none, unilateral or bilateral), and the presence of cavities by a single reader (respiratory physician 1). Cardiomegaly was defined as a CTR of >50% in a posteroanterior (PA) view and >55% in an anteroposterior view [25]. To evaluate the interobserver reliability of the CXR image findings, the external validation test dataset of 100 CXR images was independently read by respiratory physicians 1 and 2, both of whom are board-certified with more than 10 years of experience. Interobserver reliability for the interpretation of radiographic findings was assessed by calculating agreement rates and the kappa statistic (*κ*) [26]. The CXR images were de-identified and saved as Joint Photographic Experts Group files with a resolution of 720 × 960 pixels. Data augmentation techniques were not used in this study.

### 2.3. Google Cloud AutoML Vision

Google Cloud AutoML Vision is a publicly available platform that provides automated deep learning models through training, evaluation, and prediction based on images [10].

Models using Google Cloud AutoML Vision showed discriminative performance and diagnostic properties comparable to those of state-of-the-art deep learning algorithms [10]. Google Cloud AutoML Vision is used in diagnostic research using pathological and ultrasound images of breast cancer, diagnostic research using otoscopic images, research on retinal diseases, and evaluation of spermatogenesis using histological images of tests (Table S4). In this study, the original CXR image dataset was uploaded to Google Cloud storage and allocated to the training, validation, and test datasets (80%, 10%, and 10%, respectively) randomly in Google Cloud AutoML Vision. 10% of the dataset was used for validation. The model learning framework incorporates training data at each iteration of the training process and then uses the model's performance on the validation set to adjust the model's hyperparameters (variables that specify the model's structure). In the current study, we used Google Cloud AutoML Vision to create a deep learning model for classifying CXR images of fatal or nonfatal pneumonia.

### 2.4. Performance of the Deep Learning Model in External Validation and Comparison with Physicians

After training, the deep learning model using Google Cloud AutoML Vision was deployed for online predictions. The model provided a score for each prediction of pneumonia prognosis based on CXR images. The score was a confidence estimate between 0.0 and 1.0. A higher value indicated greater confidence that the annotation was accurate. We assessed the performance of the deep learning model using an external validation test dataset of 100 CXR images to verify the generalizability of the model. The external validation test dataset was not used in the training, validation, or testing of the deep learning models. In addition, respiratory physicians 2 and 3 and residents 1 and 2, who were not informed of the prognosis of pneumonia patients, were asked to infer the prognosis from the 100 CXR images of the external validation test dataset. Respiratory physician 3 is a board-certified physician with more than 10 years of experience. Residents 1 and 2 are physicians within 2 years of graduation.

### 2.5. Sony Neural Network Console

**Figure 2:**
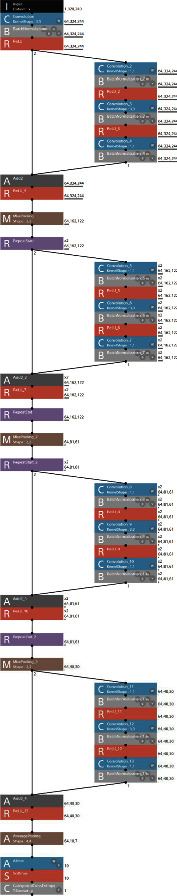
Network architectures of the ResNet model. The revised template of the network (tutorial.basics. 12_residual_learning) (https://dl.sony.com/) was used to provide the structure of the deep neural network. The square box indicates the function of the layer. The numbers to the right of the box indicate the specifications of each layer. For example, the three numbers to the right of the first input layer indicate the number of colors and size (height and width) of the input image, respectively. In the second convolutional layer, the same format is used to indicate the number of outputs and size (height and width) of the feature map, respectively. “ReLU” stands for rectified linear unit. “Kernel shape” indicates the pixel size of each filter for convolution of the input. The NNC models were trained with a batch size of 16, epochs of 100, and Adam optimization (learning rate 0.001). For other specifications, please refer to the reference (Sony Network Communications Inc. 2020).

Sony Neural Network Console (NNC) is a graphical user interface-based deep learning development tool [27]. NNC has been used in studies of retinal diseases and the classification of neutrophil fractions (Table S4). We evaluated whether NNC can also be used to create a deep learning prediction model with the ResNet model for fatal pneumonia from CXR images. We used the same dataset for training NNC and Google Cloud AutoML vision (Figure 1, Table 1). The ResNet model, shown in Figure 2, is a neural network model proposed by Microsoft Research in 2015 and is believed to exhibit high image discrimination performance [28]. In addition, the application of the deep learning model by ResNet to image diagnosis of pneumonia using CXR images had shown high performance, with a sensitivity of 96.5%, specificity of 92.7%, and accuracy of 94.6% [15], and it was expected that a high-performance model would be developed in this study.

### 2.6. Statistical Analysis

Google Cloud AutoML Vision provides an area under the precision-recall curve (AUPRC), sensitivity (recall), and positive predictive value (PPV) (precision). Sensitivity, specificity, PPV, negative predictive value (NPV), and accuracy were calculated to evaluate the performance of the model at a threshold of 0.5. In the deep learning model with NNC, the sensitivity, specificity, PPV, NPV, and accuracy were also calculated. Similar metrics were calculated for the prognostic performance of physicians on the external validation test dataset of 100 CXR images. We evaluated the performance of our deep learning models using sensitivity, specificity, PPV, negative predictive value (NPV), accuracy, and F1 score. Categorical variables were compared using Fisher's exact test. Survival was evaluated using the Kaplan–Meier method, and differences in survival were analyzed using the log-rank test. The observed proportional interobserver agreement rate for the presence or absence of radiographic findings was calculated by summation of the proportions of equal interpretations of two board-certified respiratory physicians (respiratory physicians 1 and 2). The kappa statistic is a measure of interobserver reliability that adjusts for agreement by chance. A *κ* < 0.20 indicates poor agreement; a *κ* of 0.21–0.40, fair agreement; a *κ* of 0.41–0.60, moderate agreement; a *κ* of 0.61–0.80, good agreement; and a *κ* of 0.81–1.00 indicates very good agreement between two observers [26]. Logistic regression analyses were used to examine the associations among radiographic characteristics, complications of congestive heart failure, and mortality. In the first step, each risk factor was tested individually in a univariate analysis by Fisher's exact test. In the second step, all risk factors that showed an association in the univariate model (*P* < 0.15) were added to the multivariable model. Finally, a backward stepwise selection was used to determine factors associated with mortality. All statistical analyses were performed using EZR, a graphical user interface for R [29].

## 3. Results

### 3.1. Interobserver Variation in the Interpretation of CXR Image Findings for Pneumonia

Table S3 shows the patient characteristics of the 100 patients with pneumonia prepared for the external validation of the deep learning models. Two respiratory physicians (respiratory physicians 1 and 2) evaluated the findings of these CXR images at pneumonia diagnosis.  Table S5 shows the agreement rates on the specific patterns of radiographic infiltrates in the external validation test dataset in which both respiratory physicians agreed on the presence of a pulmonary infiltrate. Among the external validation test datasets, the calculation of agreement rates and *κ* demonstrated the following results: the number of lobes involved (overall agreement, 86%; *κ* = 0.62); location of the infiltrate (overall agreement, 77%; *κ* = 0.529), pleural effusion (location) (overall agreement, 73%; *κ* = 0.687), and cavitation (overall agreement, 97%; *κ* = 0.556) (Table S5).

### 3.2. Patient Demographic Characteristics in the CXR Image Original Dataset for Training of Deep Learning Models

Of 1356 patients with pneumonia, 1274 (94.0%) were included in the present study (Figure 1(a)). The demographic and clinical characteristics of the study participants are presented in Table 1. The cohort comprised 750 (58.9%) men and 524 (41.1%) women with a median age of 75 years (range: 15–104 years). A total of 1031 (80.9%) patients had nonfatal pneumonia, and 243 (19.1%) patients had fatal pneumonia. A positive sputum culture was found in 455 (35.7%) patients with pneumonia, and the most common organism detected was methicillin-resistant *Staphylococcus aureus* (MRSA) (Table S6).

### 3.3. Association of Radiographic Findings and Cardiac Complications with Fatal Pneumonia in the Original Dataset of 1274 Patients with Pneumonia

Univariate analyses demonstrated the following radiographic characteristics to be significantly associated with fatal pneumonia (Table 2): (1) cardiomegaly (odds ratio (OR), 1.69; 95% confidence interval (CI), 1.26–2.26, *P* < 0.0005); (2) two or more lobes involved with infiltrates (OR, 15.08; 95% CI, 8.66–28.41, *P* < 0.0001); (3) bilateral infiltrate (OR, 5.25; 95% CI, 3.75–7.45, *P* < 0.0001); (4) unilateral (OR, 1.78; 95% CI, 1.22–2.59, *P* < 0.0005) or bilateral pleural effusion (OR, 4.69; 95% CI, 3.28–6.71, *P* < 0.0001) (Table 2). Furthermore, complications of CHF were observed in 9.8% of patients with pneumonia and were significantly associated with fatal pneumonia (OR, 4.68; 95% CI, 3.12–7.01, *P* < 0.0001) (Table 3). Multivariate logistic regression analysis revealed that two or more lobes involved with infiltrates (odds ratio: 11.3, 95% confidence interval: 6.39–20.00, *P* < 0.0001), no pleural effusion compared to unilateral pleural effusion (odds ratio: 0.50, 95% confidence interval: 0.35–0.73, *P* < 0.005), unilateral pleural effusion compared to bilateral pleural effusion (odds ratio: 0.53, 95% confidence interval: 0.35–0.80, *P* < 0.005), and complications of CHF (odds ratio: 3.3, 95% confidence interval: 2.17–5.01, *P* < 0.0001) were independent risk factors for mortality (Table 4).

### 3.4. Performance of the Deep Learning Model by Google Cloud AutoML Vision

**Figure 3:**
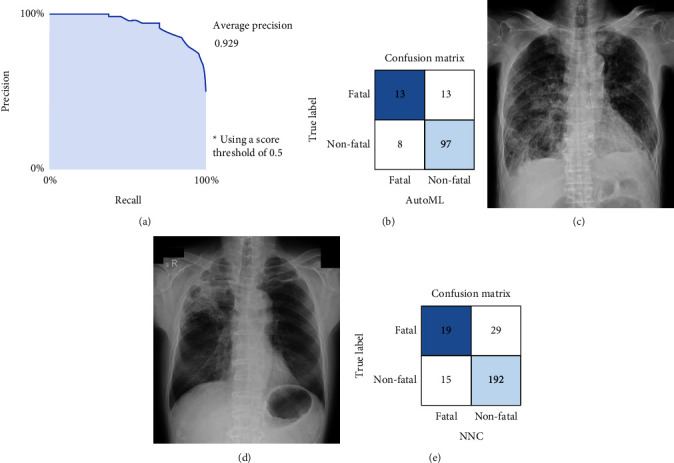
Performance results of the deep learning model by Google Cloud AutoML Vision and NNC in the original dataset for training. (a) The average precision-recall curve for all diagnoses by Google Cloud AutoML Vision. Precision is also called PPV, while recall is also called sensitivity. (b) The confusion matrix of validation results for the test data by Google Cloud AutoML Vision. (c) An example of fatal pneumonia CXR images at diagnosis predicted accurately by Google Cloud AutoML Vision. (d) An example of nonfatal pneumonia CXR images at diagnosis predicted accurately by Google Cloud AutoML Vision. (e) The confusion matrix of validation results for the test data by NNC. CXR, chest X-ray; PPV, positive predictive value.

A total of 1016 CXR images randomly selected by the platform were used for training, 125 CXR images were used for validation, and 131 CXR images were used for testing in Google Cloud AutoML Vision (Figure 1(b)). Based on the self-evaluation of the platform, the deep learning model using Google Cloud AutoML Vision showed an AUPRC of 0.929, with a sensitivity of 50.00% and specificity of 92.4%, and accuracy of 84.0% (Figure 3(a) and Table 5). The confusion matrix of validation results for the test data is shown in Figure 3(b).  Figure 3(c) shows the CXR image at pneumonia diagnosis that was correctly assessed as fatal pneumonia by the deep learning model.  Figure 3(d) shows the CXR image at pneumonia diagnosis that was correctly assessed as nonfatal pneumonia by the deep learning model.

### 3.5. External Validation and Analysis of Poor Prognostic Findings in Pneumonia CXR Images at Diagnosis by the Deep Learning Model with Google Cloud AutoML Vision

**Figure 4:**
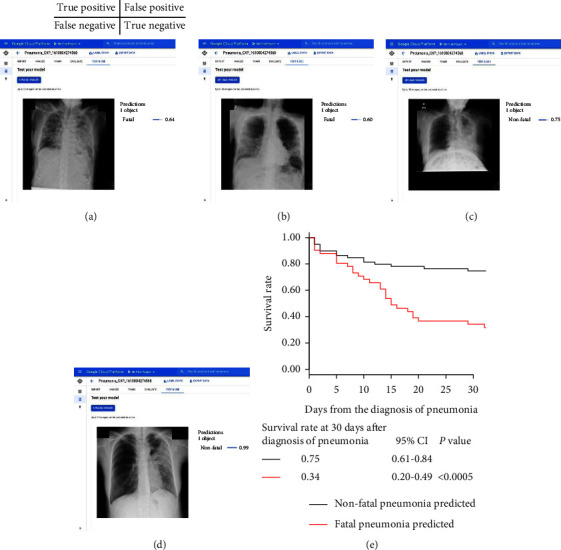
Overview of the deployed deep learning model viewer by Google Cloud AutoML Vision and Kaplan–Meier plots for time to death from the diagnosis of pneumonia in the external validation test dataset. (a) A true-positive CXR image: the deep learning model accurately predicted it as a fatal case with a score of 0.64, and the actual prognosis was fatal. (b) A false-positive CXR image: the deep learning model predicted it as a fatal case with a score of 0.60, and the actual prognosis was nonfatal. (c) A false-negative CXR image: the deep learning model predicted it as a nonfatal case with a score of 0.75, and the actual prognosis was fatal. (d) A true-negative CXR image: the deep learning model predicted it as a nonfatal case with a score of 0.99, and the actual prognosis was nonfatal. (e) The deep learning model by Google Cloud AutoML Vision predicted nonfatal or fatal pneumonia using CXR images (a survival rate of 0.34 for patients with fatal pneumonia predicted versus 0.75 for those with nonfatal pneumonia predicted, *P* < 0.0005). CXR, chest X-ray.

**Figure 5:**
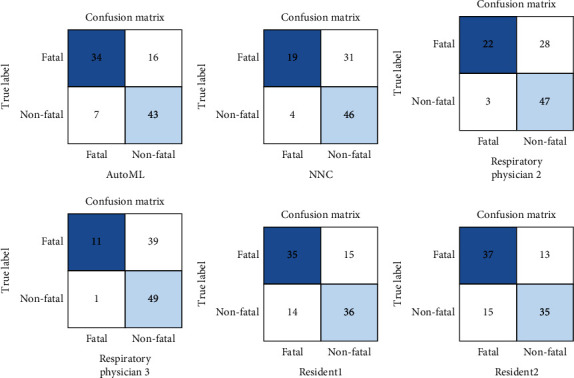
Confusion matrix of prediction performance of fatal and nonfatal pneumonia in the external validation test dataset by deep learning models and physicians.

**Figure 6:**
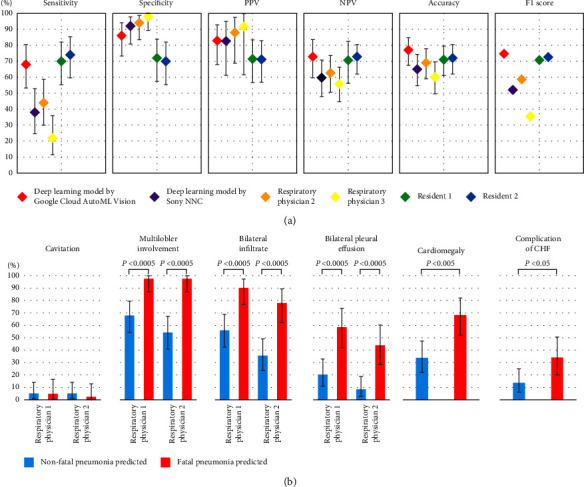
Comparison and nature of prediction performance of fatal and nonfatal pneumonia in the external validation test dataset by deep learning models and physicians. (a) Performance of the deep learning models, two board-certified respiratory physicians, and two residents based on assessments of the external validation test dataset of 100 CXR images. (b) In 100 CXR images of the test dataset for external validation, respiratory physicians 1 and 2 evaluated the cavities, the number of involved lung lobes, the location of infiltrates, and pleural effusions, respectively. The percentages of these findings were compared between the groups that the deep learning model by Google Cloud AutoML Vision predicted as nonfatal or fatal pneumonia. We also compared the rates of cardiomegaly and CHF in the group that the deep learning model by Google Cloud AutoML Vision predicted as nonfatal or fatal pneumonia. Error bars represent 95% confidence intervals. *P* values were determined by Fisher's exact test. CXR, chest X-ray; CHF, congestive heart failure.

We deployed a deep learning model using Google Cloud AutoML Vision for online predictions. An overview of the deployed deep learning model viewer is shown in Figures 4(a)–4(d). The Kaplan–Meier plots for time to death from the diagnosis of pneumonia showed that patients predicted for fatal pneumonia had a lower survival rate at 30 days after diagnosis of pneumonia than patients predicted for nonfatal pneumonia according to the prediction by the deep learning model by Google Cloud AutoML Vision in the external validation test dataset (Figure 4(e)). The performance of the deep learning model using Google Cloud AutoML Vision for classifying fatal and nonfatal pneumonia using the external validation test dataset is shown in Figure 5 (confusion matrix) and Figure 6(a), and the numerical values are presented in Table 6. In the group predicted to have fatal pneumonia by the deep learning model by Google Cloud AutoML Vision, the rate of poor prognostic findings on pneumonia CXR images and complications, such as multilobar involvement, bilateral infiltrate, bilateral pleural effusion, cardiomegaly, and complication of CHF, were significantly higher than those in the group predicted to have nonfatal pneumonia (Figure 6(b)).

### 3.6. Performance of the Deep Learning Model by NNC

The CXR images of pneumonia diagnosis were randomly allocated to the training and validation datasets (80% and 20%, respectively) (Figure 1(b)). The CXR images used in NNC were not trained at 720 × 960 pixels because of technical problems; therefore, the images were processed to 240 × 320 pixels for further training (Figure 2). An evaluation using validation data from a deep learning model by NNC showed a sensitivity of 39.6%, specificity of 92.8%, and accuracy of 82.7% (Table 5). The confusion matrix of validation results for the test data is shown in Figure 3(e). The performance of the deep learning model by NNC for classifying fatal and nonfatal pneumonia using the external validation test dataset is shown in Figure 5 (confusion matrix) and Figure 6(a), and the numerical values are presented in Table 6.

### 3.7. Comparison of the Performance between the Deep Learning Models and Physicians

Respiratory physicians had better specificity and PPV than deep learning models (Figure 6(a) and Table 6). On the other hand, residents had lower specificity and PPV than deep learning models (Figure 6(a) and Table 6).

## 4. Discussion

We developed deep learning models to predict fatal pneumonia using CXR images. The deep learning prediction models showed a performance comparable to that of physicians in predicting the prognosis of pneumonia based on CXR images (Figure 6(a) and Table 6). These results suggest that the deep learning model is useful for prognostic evaluation using CXR images in patients with pneumonia at diagnosis. Feng et al. developed a deep learning prognostic model for CAP using nonimaging data (such as comorbidities, vitals, and blood biomarkers), with a sensitivity of 74.4% to 98.2%, specificity of 83.1% to 100%, and accuracy of 79.3% to 99% [30]. Furthermore, deep learning models have been reported to predict the severity of COVID-19 pneumonia using CXR images [21–23]. However, the prognosis prediction of non-COVID-19 pneumonia by deep learning using CXR images has not been sufficiently studied. Our report suggests that AI with deep learning can also be useful in predicting the prognosis of pneumonia using CXR images with the same level of performance as the similar study above, which was innovative noticeably. Deep learning models for automated assessment of COVID-19 pneumonia severity on CXR have been trained using radiologists' CXR severity scores as labels [21, 22]. These labelings by severity scores are subjective to interpretation and variability exists [23]. On the other hand, image labeling in this study is highly objective, based on the clinical outcome data (fatal or nonfatal) which are a ground truth definition [31].

Multilobar pneumonia, bilateral pneumonia, and bilateral pleural effusions have been reported as poor prognostic factors for pneumonia [4, 5]. Similarly, these findings were also poor prognostic factors in our study (Table 2). In addition, external validation showed that these findings were significantly more frequent in the group predicted as fatal pneumonia than in the group predicted as nonfatal pneumonia by Google Cloud AutoML Vision (Figure 6(b)). These results suggest that the deep learning model may have learned these findings as features of fatal pneumonia. In this study, 9.8% of patients with pneumonia also had CHF (Table 1). It has been reported that the prognosis of pneumonia is poor in patients with CHF [24]. In this study, the multivariate logistic regression model showed that the complication of heart failure in patients with pneumonia was an independent risk factor. The risk of death in pneumonia patients with CHF was 3.3 times higher than that in pneumonia patients without CHF (Table 4). Furthermore, external validation by the deep learning model of Google Cloud AutoML Vision showed that the group predicted to have fatal pneumonia contained significantly more patients with CHF than the group predicted to have nonfatal pneumonia (Figure 6(b)). This result suggests that the deep learning model can accurately differentiate between fatal and nonfatal pneumonia, even in pneumonia patients with CHF.

The performance evaluation of deep learning using the Google Cloud AutoML Vision model in differentiating fatal pneumonia from the external validation test dataset showed a sensitivity of 68%, specificity of 86%, and accuracy of 77% (Figure 6(a) and Table 6). The sensitivity and accuracy of NNC were lower than those of Google Cloud AutoML Vision, but the specificity was as high as 92.0%. This may have been due to the effect of image degradation during training and the small number of fatal cases. The CXR images used for NNC could not be trained at 720 × 960 pixels due to technical problems, so images processed to 240 × 320 pixels were used for training. In addition, in the case of Google Cloud AutoML, the details of the architecture of the model are not known, making it difficult to study the details, which is an issue that needs to be considered in the future. There is a good possibility that the performance of deep learning models can be improved by increasing the number of training data. Further study of additional metadata such as age, gender, and presence/absence of heart failure complications is expected to further improve learning performance and is considered a topic for future research. Furthermore, in terms of specificity and PPV, the performance of both deep learning models on the two platforms was comparable to that of the physicians. These results indicated that the reproducibility of deep learning pneumonia prognosis modeling using CXR images had good performance. Additionally, the accuracy and F1 score of the deep learning model using Google Cloud AutoML Vision were higher than those of board-certified respiratory physicians. These results suggest the possibility that by further improving the performance of this deep learning model, the clinical implementation of this model for the severity assessment of pneumonia patients may assist physicians in general practice, especially physicians in clinics or remote islands and suburbs, where it is difficult to consult respiratory specialists.

Compared to classical deep learning frameworks, it has been reported that the image learning performance with Google Cloud AutoML is comparable to that of conventional deep learning models [10]. What is important in the future is how to implement deep learning models in clinical practice. This study was conducted solely by clinicians, and we believe that this research is very important for the future application of deep learning models by clinicians in clinical practice.

Regarding the difference in sensitivity between respiratory physicians and residents, we were not allowed in this study to review patient history or previous examinations that have been shown to improve the physician's diagnostic ability in interpreting CXR images [32]. In particular, respiratory physicians were more likely to refer to patient history and previous examinations, which may have influenced the difference in sensitivity with residents.

In our study, 67.3% of cases were of pneumonia other than CAP (NHCAP, HAP, and VAP) (Table 1), and MRSA and *Pseudomonas aeruginosa* were reported frequently as causative organisms (Table S6). This was because most of our patients were elderly people in nursing homes, and the absolute number of NHCAP and HAP was particularly high compared to that of CAP.

This study had several limitations. First, this was a single-center study with small datasets, and these deep learning models cannot be directly applied clinically in medical institutions nationwide. Furthermore, deep learning models with higher accuracy are required for clinical applications. To create deep learning models with higher accuracy and robustness that can be used at multiple institutions, it is necessary to develop models using a larger sample size with multi-institutional data. Second, the CXR radiographic findings of the original 1274 CXR image dataset (Table 2) were assessed by a single physician (respiratory physician 1). Therefore, radiographic findings may not be sufficiently accurate [33, 34]. However, the validation using external validation data showed moderate to good agreement, with *κ* values ranging from 0.529 to 0.687 between respiratory physicians 1 and 2 (Table S5). Furthermore, the performance evaluation of the deep learning model in the external validation showed a similar trend in the radiographic findings assessed by respiratory physicians 1 and 2 (Figure 6(b)). Based on these results, the radiologic findings in the original 1274 CXR image dataset at pneumonia diagnosis were also considered to have a certain degree of accuracy. Third, the model cannot retain its ability to accurately diagnose fatal pneumonia without updating. Medical care is advancing daily, and the survival rate of pneumonia is also expected to change over time. Therefore, deep learning models must be retrained using additional data to dynamically update their performance [35].

## 5. Conclusions

The diagnostic tool based on deep learning models yielded good classification accuracy for classifying fatal pneumonia. By further improving the performance of these learning models, AI could assist physicians in the severity assessment of pneumonia patients in general practice.

## Figures and Tables

**Table 1:** Characteristics of the study patients in the CXR image original dataset for training of deep learning models (*n* = 1274).

Characteristics	*N*	(%)
Median age (range), years	75 (15–104)	
Gender		
Male	750	58.9
Female	524	41.1

Hospitalization for pneumonia treatment		
No	282	22.1
Yes	992	77.9

Pneumonia type		
CAP	417	32.7
Other than CAP (NHCAP, HAP, VAP)	857	67.3

Complication of congestive heart failure		
No	1149	90.2
Yes	125	9.8

Positive results with the sputum culture test	455	35.7
Positive results with the blood culture test	20	1.6
Positive results with the pleural fluid culture test	4	0.3
Positive results with the bronchial lavage fluid culture test	7	0.5
Posteroanterior chest radiographs	841	66.0
Chest radiographs under intubation	15	1.2

Prognosis		
Nonfatal	1031	80.9
Fatal	243	19.1

CAP, community-acquired pneumonia; NHCAP, nursing and healthcare-associated pneumonia; HAP, hospital-acquired pneumonia; VAP, ventilator-associated pneumonia.

**Table 2:** Univariate association of radiographic characteristics and mortality in the original dataset (*n* = 1274).

Radiographic characteristics	Frequency, no, (%)	Mortality rate (%)	OR (95% CI)	*P* value
Cardiomegaly				
No	711 (55.8)	15.5		^…a^
Yes	563 (44.2)	23.6	1.69 (1.26–2.26)	**<0.0005**

Number of lobes involved with infiltrates				
1	509 (40.0)	2.8		^…a^
≥2	765 (60.0)	29.9	15.08 (8.66–28.41)	**<0.0001**

Location of infiltrates				
Unilateral	666 (52.3)	8.0		^…a^
Bilataral	608 (47.7)	31.2	5.25 (3.75–7.45)	**<0.0001**

Pleural effusion (location)				
None	773 (60.7)	12.5		^…a^
Unilateral	280 (22.0)	20.4	1.78 (1.22–2.59)	**<0.005**
Bilateral	221 (17.3)	40.3	4.69 (3.28–6.71)	**<0.0001**

Cavitation				
No	1237 (97.1)	18.8		^…a^
Yes	37 (2.9)	27.0	1.60 (0.68–3.46)	0.21

Univariate analysis was performed by Fisher's exact test. *P* values of <0.05 are shown in bold. ^a^ellipses indicate that this variable was used as the baseline variable in the univariate analysis.

**Table 3:** Univariate association of complications of congestive heart failure and mortality in the original dataset (*n* = 1274).

Complication	Frequency, no, (%)	Mortality rate (%)	OR (95% CI)	*P* value
Congestive heart failure				
No	1149 (90.2)	16.0		^…a^
Yes	125 (9.8)	47.2	4.68 (3.12–7.01)	**<0.0001**

Univariate analysis was performed by Fisher's exact test. *P* values of <0.05 are shown in bold. ^a^ellipses indicate that this variable was used as the baseline variable in the univariate analysis.

**Table 4:** Multivariate analysis.

Factors	OR (95% CI)	*P* value
Number of lobes involved with infiltrates		
1	1.0	
≥2	11.3 (6.39–20.00)	**<0.0001**

Pleural effusion (location)		
None	0.50 (0.35–0.73)	**<0.005**
Unilateral	1.0	

Pleural effusion (location)		
Unilateral	0.53 (0.35–0.80)	**<0.005**
Bilateral	1.0	

Congestive heart failure		
No	1.0	^…a^
Yes	3.3 (2.17–5.01)	**<0.0001**

P values of <0.05 are shown in bold.

**Table 5:** The performance of the deep learning models in the original dataset for training.

	AUPRC	Sensitivity (%)	Spcecificity (%)	PPV (%)	NPV (%)	Accuracy (%)
AutoML	0.929	50.0	92.4	61.9	88.2	84.0
NNC	NR	39.6	92.8	55.9	86.9	82.7

NR, not reported.

**Table 6:** Performance measures of the deep learning models and physicians on the external validation test dataset.

	AutoML	NNC	Respiratory physician 2	Respiratory physician 3	Resident 1	Resident 2
Sensitivity (95% CI)	68.0 (53.3–80.5)	38.0 (24.7–52.8)	44.0 (30.0–58.7)	22.0 (11.5–36.0)	70.0 (55.4–82.1)	74.0 (59.7–85.4)

Specificity (95% CI)	86.0 (73.3–94.2)	92.0 (80.8–97.8)	94.0 (83.5–98.7)	98.0 (89.4–99.9)	72.0 (57.5–83.8)	70.0 (55.4–82.1)

PPV (95% CI)	82.9 (67.9–92.8)	82.6 (61.2–95.0)	88.0 (68.8–97.5)	91.7 (61.5–99.8)	71.4 (56.7–83.4)	71.2 (56.9–82.9)

NPV (95% CI)	72.9 (59.7–83.6)	59.7 (47.9–70.8)	62.7 (50.7–73.6)	55.7 (44.7–66.3)	70.6 (56.2–82.5)	72.9 (62.1–80.5)

Accuracy (95% CI)	77.0 (67.5–84.8)	65.0 (54.8–74.3)	69.0 (59.0–77.9)	60.0 (49.7–69.7)	71.0 (61.1–79.6)	72.0 (62.1–80.5)

F1 score (%)	74.7	52.1	58.7	35.5	70.7	72.6

CI, confidence interval.

## Data Availability

The dataset is not publicly available for legal and ethical reasons.

## Abbreviations

AI: Artificial intelligence
AP: Anteroposterior
AUPRC: Area under the precision-recall curve
BNP: Brain natriuretic peptide
CAP: Community-acquired pneumonia
CHF: Congestive heart failure
COVID-19: Coronavirus disease 2019
CTR: Cardiothoracic ratio
CXR: Chest X-ray
HAP: Hospital-acquired pneumonia
JPEG: Joint Photographic Experts Group
LVEF: Left ventricular ejection fraction
MRSA: Methicillin‐resistant *Staphylococcus aureus*
NHCAP: Nursing and healthcare-associated pneumonia
NPV: Negative predictive value
OR: Odds ratio
PA: Posteroanterior
PPV: Positive predictive value.

## Supplementary Materials

Supplementary Materials Supplementary Table S1. Diagnostic criteria for pneumonia.
