# Supplementary material for: Deep Learning Models to Predict Fatal Pneumonia Using Chest X-Ray Images
Source: Can Respir J. 2022 Nov 24;2022:8026580. doi: 10.1155/2022/8026580 (PMC9715345; doi:10.1155/2022/8026580)
Supplement: Supplementary Materials — Supplementary Table S1. Diagnostic criteria for pneumonia. [file 8026580.f1.docx]

**Supplementary Table S1. Diagnostic criteria for pneumonia**

| **Symptomatic criteria**  (1) Fever (body temperature of ≥ 37.0℃) or cough or dyspnea  (2) Leukocytosis (WBCs >10,000/μL) or leukopenia (WBCs <4,000/μL) or high CRP levels (0.30 mg/dL)  (3) Worsening of hypoxemia (SpO_2_ ≤90%, or PaO_2_ ≤60 Torr) |
| --- |
| **Radiological criteria**  (1) Chest x-ray suggestive of pneumonia  (2) Chest CT scans suggestive of pneumonia |

Note: The each criteria must meet at least one factor from each items. The patients that met symptomatic and radiological criteria were designated as a case of pneumonia.

Abbreviations not defined in text: WBCs, white blood cells; CRP, C-reactive protein.

**Supplementary Table S2. Diagnostic criteria for new heart failure**

| **(i)** The simultaneous presence of clinical signs of new pulmonary edema or acute CHF (rales, increased jugular venous pressure, S3 gallop, peripheral edema) detected by the managing physician on physical examination and documented in the medical record. |
| --- |
| **(ii)** A plasma brain natriuretic peptide (BNP) level ≥ 200 pg/mL at admission or echocardiography shows decreased contractility {left ventricular ejection fraction (LVEF) (modified Simpson method) < 50%} or decreased diastolic capacity. |
| **(iii)** Unplanned addition of oral or intravenous loop diuretic drugs, dose-up of oral loop diuretics, addition of a thiazide diuretic drug to loop diuretics, or the need for treatment with intravenous vasodilators and intravenous inotropes. |

Note: The each criteria (i-iii) must meet at least one factor from each items. The patients that met each criteria were designated as a case of new heart failure.

**Supplementary Table S3. Characteristics of the study patients for the**

**external validation test dataset (n = 100)**

| Characteristic | n | % |
| --- | --- | --- |
| Median age (range), years | 78 (21 - 100) | |
| Gender |  | |
| Male | 58 | 58.0 |
| Female | 42 | 42.0 |
| Hospitalization for pneumonia treatment |  |  |
| No | 25 | 25.0 |
| Yes | 75 | 75.0 |
| Pneumonia type |  |  |
| CAP | 28 | 28.0 |
| Other than CAP (NHCAP, HAP, VAP) | 72 | 72.0 |
| Complication of congestive heart failure |  |  |
| No | 78 | 78.0 |
| Yes | 22 | 22.0 |
| Positive results with sputum culture test | 49 | 49.0 |
| Positive results with blood culture test | 2 | 2.0 |
| Positive results with pleural fluid culture test | 1 | 1.0 |
| Positive results with bronchial lavage fluid culture test | 1 | 1.0 |
| Posteroanterior chest radiographs | 43 | 43.0 |
| Chest radiographs under intubation | 3 | 3.0 |
| Prognosis |  |  |
| Non-fatal | 50 | 50.0 |
| Fatal | 50 | 50.0 |

Abbreviations not defined in text: CAP, Community-acquired pneumonia; NHCAP, Nursing and

healthcare-associated pneumonia; HAP, Hospital-acquired pneumonia; VAP, Ventilator-associated pneumonia.

**Supplementary Table S4.**

**Studies using Google Cloud AutoML Vision in clinical medicine**

| **Author** | **year** | **Journal** | **Image data for training of deep learning model** | **Advantages demonstrated by deep learning models** |
| --- | --- | --- | --- | --- |
| Faes L^1^ | 2019 | Lancet Digital Health | Five publicly available open-source datasets: retinal fundus images; optical coherence tomography images; images of skin lesions, and both paediatric and adult chest x-ray images | Almost models showed comparable discriminative performance and diagnostic properties to state-of-the-art performing deep learning algorithms. |
| Zeng Y^2^ | 2020 | Comput Biol Med | 378,215 histopathology images of Invasive ductal carcinoma of breast cancer | Invasive ductal carcinoma of breast cancer can be automatically diagnosed based on histopathology images. |
| Livingstone D^3^ | 2020 | Laryngoscope | 1366 otoscopic images | Fourteen different otologic diagnoses can be made automatically. |
| Wan KW^4^ | 2021 | Quant Imaging Med Surg | 895 breast ultrasound images | Benign and malignant breast lesions can be automatically diagnosed based on breast ultrasound images. |
| Kim IK^5^ | 2021 | Br J Ophthalmol | 783 ultra-widefield indocyanine green angiography images of patients with pachychoroid and non-pachychoroid diseases | Classification of pachychoroid disease on ultrawide-field indocyanine green angiography can be made automatically. |
| Ito Y^6^ | 2021 | Sci Rep | 7155 histopathology images of testis with seminiferous tubules | Histopathological evaluation of the testis in spermatogenesis can be made automatically. |

**Studies using Sony Neural Network Console in clinical medicine**

| **Author** | **year** | **Journal** | **Image data for training of deep learning model** | **Advantages demonstrated by deep learning models** |
| --- | --- | --- | --- | --- |
| Aoyama Y^7^ | 2021 | PLoS One | 80 en face images of the choroidal vasculature of the segmentation slab of one-half of the subfoveal choroidal thickness | Central serous chorioretinopathy can be automatically diagnosed by deep learning with high accuracy from en face images of the choroidal vasculature. |
| Yabuta M^8^ | 2021 | Tohoku J Exp Med | 600 images of neutrophils | Neutrophils can be automatically differentiated based on their count of segmented nuclei using deep learning. |

**Supplementary Table S5. Agreement on the pattern of radiographic pulmonary infiltrate by two**

**respiratory physicians in the external validation test dataset (n = 100)**

| Radiographic characteristics | Respiratory physician 1 | Respiratory physician 2 | Overall agreement (%) | Kappa | *P* value |
| --- | --- | --- | --- | --- | --- |
| Number of lobes involved |  |  | 86/100 (86) ^a^ | 0.62 | **<0.0001** |
| 1 | 20 | 28 |  |  |  |
| ≥ 2 | 80 | 72 |  |  |  |
| Location of infiltrate |  |  | 77/100 (77) ^b^ | 0.529 | **<0.0001** |
| Unilateral | 30 | 47 |  |  |  |
| Bilataral | 70 | 53 |  |  |  |
| Pleural effusion (location) |  |  | 73/100 (73) ^c^ | 0.687 | **<0.0001** |
| None | 43 | 56 |  |  |  |
| Unilateral | 21 | 21 |  |  |  |
| Bilateral | 36 | 23 |  |  |  |
| Cavitation |  |  | 97/100 (97) ^d^ | 0.556 | **<0.0001** |
| No | 95 | 96 |  |  |  |
| Yes | 5 | 4 |  |  |  |

*P* values were determined with Fisher’s exact test, and those of < 0.05 are shown in bold.

^a^ Overall agreement on the number of lobes involved was defined as the sum of the proportion of radiographs read as unilobar (17/100) and as multilobar (69/100) by both respiratory physicians.

^b^ Overall agreement on the location of infiltrate was defined as the sum of the proportion of radiographs read as unilateral (27/100) and as bilateral (50/100) by both respiratory physicians.

^c^ Overall agreement on the pleural effusion (location) was defined as the sum of the proportion of radiographs read as none (40/100) and as unilateral (11/100) and as bilateral (22/100) by both respiratory physicians.

^d^ Overall agreement on the cavitation was defined as the sum of the proportion of radiographs read as no (94/100) and as yes (3/100) by both respiratory physicians.

**Supplementary Table S6. Detected bacteria in the original dataset (n=929)**

| Bacteria | n | % |
| --- | --- | --- |
| *Streptococcus pneumonia* | 39 | 4.2 |
| *MSSA* | 84 | 9.0 |
| *MRSA* | 101 | 10.9 |
| *Streptococci other than Streptococcus pneumonia* | 22 | 2.4 |
| *Pseudomonas aeruginosa* | 71 | 7.6 |
| *Klebsiella spp.* | 57 | 6.1 |
| *Haemophilus influenzae* | 57 | 6.1 |
| *Haemophilus parainfluenzae* | 23 | 2.5 |
| *Enterobacter spp.* | 17 | 1.8 |
| *Escherichia coli* | 50 | 5.4 |
| *Serratia spp.* | 7 | 0.8 |
| *Stenotrophomonas maltophilia* | 13 | 1.4 |
| *Acinetobacter spp.* | 14 | 1.5 |
| *Citrobacter spp.* | 7 | 0.8 |
| *Moraxella catarrhalis* | 13 | 1.4 |
| *Proteus spp.* | 2 | 0.2 |
| Other organisms | 37 | 3.9 |

Abbreviations not defined in text: MSSA, methicillin-sensitive *Staphylococcus aureus*.

**REFERENCES**

1. Faes L, Wagner SK, Fu DJ, et al. Automated deep learning design for medical image classification by health-care professionals with no coding experience: a feasibility study. *The Lancet Digital Health*. 2019;1(5): e232-e242. https://doi.org/10.1016/s2589-7500(19)30108-6.

2. Zeng Y, Zhang J. A machine learning model for detecting invasive ductal carcinoma with Google Cloud AutoML Vision. *Comput Biol Med*. 2020;122: 103861. https://doi.org/10.1016/j.compbiomed.2020.103861.

3. Livingstone D, Chau J. Otoscopic diagnosis using computer vision: An automated machine learning approach. *Laryngoscope*. 2020;130(6): 1408-1413. https://doi.org/10.1002/lary.28292.

4. Wan KW, Wong CH, Ip HF, et al. Evaluation of the performance of traditional machine learning algorithms, convolutional neural network and AutoML Vision in ultrasound breast lesions classification: a comparative study. *Quant Imaging Med Surg*. 2021;11(4): 1381-1393. https://doi.org/10.21037/qims-20-922.

5. Kim IK, Lee K, Park JH, Baek J, Lee WK. Classification of pachychoroid disease on ultrawide-field indocyanine green angiography using auto-machine learning platform. *Br J Ophthalmol*. 2021;105(6): 856-861. https://doi.org/10.1136/bjophthalmol-2020-316108.

6. Ito Y, Unagami M, Yamabe F, et al. A method for utilizing automated machine learning for histopathological classification of testis based on Johnsen scores. *Sci Rep*. 2021;11(1): 9962. https://doi.org/10.1038/s41598-021-89369-z.

7. Aoyama Y, Maruko I, Kawano T, et al. Diagnosis of central serous chorioretinopathy by deep learning analysis of en face images of choroidal vasculature: A pilot study. *PLoS One*. 2021;16(6): e0244469. <https://doi.org/10.1371/journal.pone.0244469>.

13. Yabuta M, Nakamura I, Ida H, et al. Deep Learning-Based Nuclear Lobe Count Method for Differential Count of Neutrophils. *Tohoku J Exp Med*. 2021;254(3): 199-206. https://doi.org/10.1620/tjem.254.199.
